# The MAP kinase *HwHog1 *from the halophilic black yeast *Hortaea werneckii*: coping with stresses in solar salterns

**DOI:** 10.1186/1746-1448-3-3

**Published:** 2007-03-09

**Authors:** Metka Lenassi, Tomaz Vaupotic, Nina Gunde-Cimerman, Ana Plemenitas

**Affiliations:** 1 Institute of Biochemistry, Faculty of Medicine, University of Ljubljana, Vrazov trg 2, Ljubljana, Slovenia; 2 Biology Department, Biotechnical Faculty, University of Ljubljana, Vecna pot 111, Ljubljana, Slovenia

## Abstract

**Background:**

*Hortaea werneckii *is one of the most salt-tolerant species among microorganisms. It has been isolated from hypersaline waters of salterns as one of the predominant species of a group of halophilic and halotolerant melanized yeast-like fungi, arbitrarily named as "black yeasts". It has previously been shown that *H. werneckii *has distinct mechanisms of adaptation to high salinity environments that are not seen in salt-sensitive and only moderately salt-tolerant fungi. In *H. werneckii*, the HOG pathway is important for sensing the changes in environmental osmolarity, as demonstrated by identification of three main pathway components: the mitogen-activated protein kinase (MAPK) HwHog1, the MAPK kinase HwPbs2, and the putative histidine kinase osmosensor HwHhk7.

**Results:**

In this study, we show that the expression of *HwHOG1 *in salt-adapted cells depends on the environmental salinity and that *HwHOG1 *transcription responds rapidly but reciprocally to the acute hyper-saline or hypo-saline stress. Molecular modelling of HwHog1 reveals an overall structural homology with other MAPKs. HwHog1 complements the function of ScHog1 in the *Saccharomyces cerevisiae *multistress response. We also show that hyper-osmolar, oxidative and high-temperature stresses activate the HwHog1 kinase, although under high-temperature stress the signal is not transmitted via the MAPK kinase Pbs2. Identification of *HOG1*-like genes from other halotolerant fungi isolated from solar salterns demonstrates a high degree of similarity and excellent phylogenetic clustering with orthologues of fungal origin.

**Conclusion:**

The HOG signalling pathway has an important role in sensing and responding to hyper-osmolar, oxidative and high-temperature stresses in the halophilic fungi *H. werneckii*. These findings are an important advance in our understanding of the HOG pathway response to stress in *H. werneckii*, a proposed model organism for studying the salt tolerance of halophilic and halotolerant eukaryotes.

## Background

Solar salterns represent an extreme environment, with high concentrations of NaCl, occasional rapid changes in water activity, low oxygen concentrations and high UV radiation [1]. These environments are inhabited mostly by bacteria and archea, but also by certain algal and fungal species [2-4]. These extremophilic fungi are represented by a group of melanized fungi, the so-called black yeasts *Hortaea werneckii*, *Phaeotheca triangularis *and *Aureobasidium pullulans*, and by the non-melanized fungi *Wallemia sebi*, *W. muriae *and *W. ichthyophaga*. These fungi form a new group of eukaryotic halotolerant and halophilic fungi, with *H. werneckii *(*Dothideales*, *Ascomycota*) being the predominant species. *H. werneckii *is also the prevailing species at environmental salinities above 3.5 M NaCl [3].

The ability of *H. werneckii *to cope with extreme changes in environmental salinity is also demonstrable under laboratory conditions, with a defined range of growth in batch cultures from 0.0 M NaCl to saturated salinity (5.5 M NaCl) in the media, and a broad growth optimum from 1.0 M to 3.0 M NaCl. High adaptability and salt-tolerance are properties that make *H. werneckii *a highly appropriate model system for studying salt tolerance in eukaryotes [5]. It has previously been shown that *H. werneckii *has distinct mechanisms of adaptation to high-salinity environments that are not seen in salt-sensitive and only moderately salt-tolerant fungi [6]. The most relevant differences studied to date are in plasma membrane composition and properties [7], osmolyte composition and accumulation [5,8], and the maintenance of low intracellular potassium and sodium ion levels [9].

Multiple signalling pathways allow organisms to respond to different extracellular stimuli and to adjust their cellular machinery to changes in their environment. The sensing of environmental osmolarity changes is vital for cell survival. In *Saccharomyces cerevisiae*, the pathway for the sensing of these changes and responding to them is known as the high osmolarity glycerol (HOG) signalling pathway, one of the best understood mitogen-activated protein kinase (MAPK) cascades. MAPK cascades are ubiquitous in eukaryotic organisms and are composed of three sequentially acting kinases. In response to an extracellular stimulus, a MAPK kinase kinase (MAPKKK) phosphorylates and activates a MAPK kinase (MAPKK); this then phosphorylates and activates a MAPK. In the case of the HOG pathway, upon osmotic stress, the transmembrane osmosensors Sho1 and Sln1 stimulate this pathway through two distinct mechanisms, each of which leads to the activation of the key MAPK Hog1. Together with Ypd1 and Ssk1, the histidine kinase Sln1 forms a phospho-relay cascade, which transfers the signal to the MAPKK Pbs2 and its downstream MAPK, Hog1. Alternatively, Pbs2 can be activated through the Sho1-Ste20-Ste11 branch (reviewed in [10-12]). Phosphorylated Hog1 controls the transcription of a family of osmo-responsive genes, including glycerol phosphate dehydrogenase [13-15]. Although this Sln1 and Sho1 stimulation is redundant for promoting growth on high-osmolarity media, the Sln1 pathway is specialized for responses to conditions of modest osmolarity [16].

The HOG pathway has classically been considered as specific to osmotic stress, although recent studies have revealed new functions for this MAPK route. Evidence suggests that heat shock can activate Hog1 through the osmosensor Sho1 [17]. Alternatively, Sln1 can be activated by a drop in temperature [18] or by citric-acid stress [19]. It has also been shown that basal activity through the HOG pathway contributes to the regulation of cell-wall composition [20]. Additional functions have been identified for HOG-homologous pathways in other species. In *Candida albicans*, Hog1 has been implicated in the response to oxidative stress through the Sln1 branch. In the fission yeast *Schizosaccharomyces pombe*, the Sty1 pathway drives the transcriptional response to stimuli like osmotic stress, heat shock, cold stress, and oxidative and UV injury (reviewed [21]).

In *H. werneckii*, the presence of the HOG pathway was demonstrated by identification of homologous genes of the main components of the pathway: the MAPK HwHog1 [22], the MAPKK HwPbs2 and a putative histidine kinase osmosensor HwHhk7 (our unpublished data). Although the HwHog1 protein shows high homology to *S. cerevisiae *Hog1, important differences in both activation and localization of the phosphorylated and non-phosphorylated forms of HwHog1p have been seen [22]. The present study was aimed at further defining the role of HwHog1 in response to stress, by using functional complementation of *HwHOG1 *genes in *S. cerevisiae *mutants (Δ*hog1*, Δ*pbs2*) and by exposing the transformants to various stresses. We hypothesized that the HOG pathway of *H. werneckii *mediates not only high-osmolarity signals, but also responses to other environmental stresses, such as UV irradiation, high temperature, high pH, and oxidative stress, all typical of saline environments. Here, we present evidence confirming the involvement of the HOG pathway in the multi-stress response of *H. werneckii*.

## Results and Discussion

### Expression of *HwHOG1 *in salt-adapted cells depends on environmental salinity and responses to saline stress

**Figure 1:**
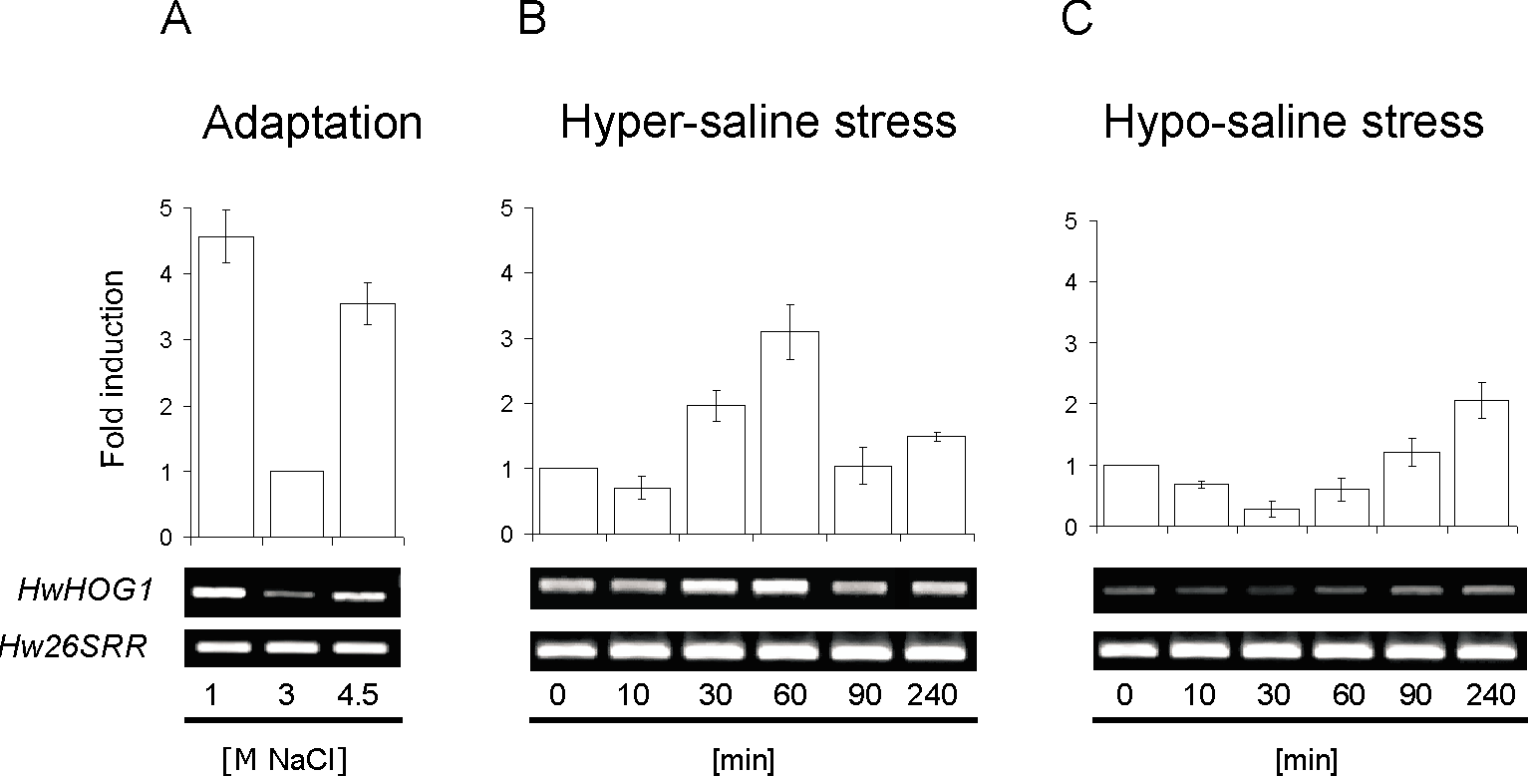
***HwHOG1 *expression in adaptation and under stress conditions**. Expression analysis of *HwHOG1 *in salt-adapted (**A**, Adaptation) and NaCl-stressed (**B**, Hyper-saline stress; **C**, Hypo-saline stress) *H. werneckii *cells at the indicated NaCl concentrations and time points. *Hw26SRR *represents a housekeeping gene for the 26S rRNA used as an internal control. The relative fold-inductions of *HwHOG1 *on cDNA levels are expressed as means (± standard deviations) of three independent experiments relative to 3.0 M NaCl in adapted cells, or relative to the time point 0 min in stressed cells.

In a previous study by Turk et al. [22], the *H. werneckii *Hog1 homologue protein (HwHog1) was identified. An *in vitro *kinase assay demonstrated that in contrast to *S. cerevisiae*, where Hog1 is activated even at very low salt concentrations, HwHog1 is fully active only at extremely high salt concentrations. In the present study, we examined how different NaCl concentrations in the growth media affect the expression of the *HwHOG1 *gene in cells adapted to 1.0 M, 3.0 M and 4.5 M NaCl and in salt-stressed cells. The transcript levels of *HwHOG1 *were examined by reverse transcription (RT)-PCR. As shown in Figure 1A, the transcript levels of *HwHOG1 *show a U-shaped profile, with approximately 5-fold and 4-fold induction at 1.0 M and 4.5 M NaCl, respectively, compared to the low level of gene expression seen at the moderate salinity of 3.0 M NaCl, which has been previously reported to be the optimal physiological condition for *H. werneckii *[5]. The transcriptional response of *HwHOG1 *to acute salt stress was relatively fast, reaching the first 2-fold change in transcript levels after 30 min, for both hyper-saline (3.0 M → 4.5 M NaCl; Fig. 1B) and hypo-saline (3.0 M → 1.0 M NaCl; Fig. 1C) stress. After the acute hyper-saline stress, the highest *HwHOG1 *transcript levels were seen after 60 min, showing an approximately 3-fold induction, as compared to the basal level of the transcript; this then rapidly declined. After the acute hypo-saline stress, the lowest *HwHOG1 *transcript levels were seen after 30 min, showing an approximately 2.5-fold repression. Following the first acute stress response for both of these stresses, a slow but continous increase in *HwHOG1 *transcript levels was seen after 90 min, suggesting the starting phase of the adaptive response, which probably contributes to the levels of the transcript seen in the adaptation study (Fig. 1A). Reciprocal transcriptional response of *HwHOG1 *as seen from the results of hypo/hyper-saline stress experiments could be connected with the action of the general stress response elements (STRE), previously identified in the promoter region of *HwHOG1 *[22]. Hog1 is regulated at different levels in different organisms. While in *S. cerevisiae *the activation of Hog1 is regulated at a posttranscriptional level [23], in *Aspergillus nidulans*, *HOG1 *gene is upregulated with exposure to high concentrations of salt [24]. In *H. werneckii*, the salt-responsive regulation of HwHog1 appeared to be at both levels, posttranslationally by phosphorylation [22], and as seen in the present study, at the level of transcription.

### Molecular modelling of HwHog1 reveals an overall structural homology with the MAPK fold

The *HwHOG1 *open reading frame (ORF) encodes a 359-amino-acid protein with a predicted molecular weight of 46 kDa and with all of the conserved regions that are specific for the MAPKs, such as the common docking (CD) domain at the C-terminal end, a TGY phosphorylation motif at amino-acid residues 171–173, and an Asp in the active site [22].

**Figure 2:**
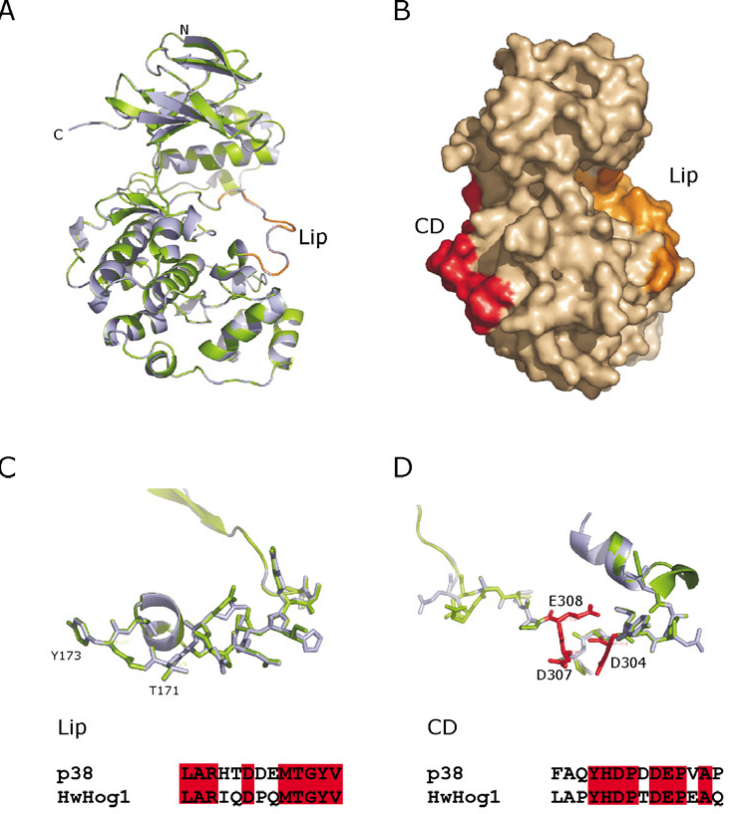
**Three-dimensional model of HwHog1**. Superposition of HwHog1 (green) and murine p38 (blue). **(A) **Ribbon diagram of the conservation of secondary structure elements. The position of the phosphorylation Lip region of HwHog1 is shown (orange). **(B) **Surface view of the HwHog1 protein. The conserved CD domain (red) and the phosphorylation Lip (orange) are exposed on the surface at opposite sides of the protein. **(C) **The architecture of the phosphorylation Lip, and **(D) **the CD domain amino-acid residues are represented by sticks. The positions of the phosphorylation site residues Thr171 and Tyr173 are marked. The negatively charged Asp304, Asp307 and Glu308 amino acids in the CD domain of HwHog1 are denoted by red sticks. The amino-acid residue conservation between the HwHog1 and p38 sequences is denoted by red boxes. The plots were created with the PyMOL programme (version 0.97 [35]).

To further examine the possible rearrangements of these key structural elements of HwHog1p, the three-dimensional (3D) model of the full-length HwHog1 protein was calculated using the MODELLER programme [25], based on crystallographic structure of murine p38 MAPK as a template [26], a functional and structural homologue of *S. cerevisiae *Hog1. This approach to comparative protein modelling was based on the satisfaction of spatial restraints [27] implemented in the MODELLER programme. Since the template structure for homology-based modelling was more than 40% identical to the sequence of the target (47.43% homology), the model created should have about 90% of the main-chain atoms comparable with the X-ray structure [25]. This calculated 3D model of the structure of HwHog1 demonstrated the well defined overall fold of the MAP kinase superfamily. The superposition of the HwHog1 model on the murine p38 template revealed a strong overlap, particularly within the conserved regions and with the active-site amino-acid residues of the phosphorylation "Lip" (Fig. 2A). Compared to the template structure, the topologically less overlapping region is a portion in the N-terminal domain that consists mainly of β-sheets. The phosphorylation Lip and the docking groove (CD domain) are exposed on opposite sides of the protein surface (Fig. 2B). The phosphorylation Lip in HwHog1 (LARIQDPQMTGYV) consists of 13 amino-acid residues (Fig. 2C), exactly as does the Lip in p38 (LARHTDDEMTGYV). The structural positioning of the critical phosphorylation residues of Thr171 and Tyr173 within the Lip of HwHog1 shows complete steric agreement with Thr180 and Tyr182 of p38, although these Lip regions share only 69% amino-acid identity. There is also an excess net negative charge in the p38 Lip as compared with the HwHog1 Lip, due to the absence of glutamate and aspartate in this region of HwHog1. Although the HwHog1 contains a proline residue in the phosphorylation Lip, this does not disturb the architecture of the Lip. Therefore, the phosphorylation site sequence of Thr171-Gly172-Tyr173 makes the essential surface turn.

The architecture of the CD domains of the MAPK docking grooves is structurally less conserved between HwHog1 (LAPYHDPTDEPEAQ) and p38 (FAQYHDPDDEPVAP), most probably due to the additional proline residue in HwHog1 (Fig. 2D). However, the three negatively charged amino-acid residues (Asp304, Asp307 and Glu308 in HwHog1) that are critical for docking interactions in p38 are structurally completely aligned between these MAP kinases.

### HwHog1 complements ScHog1 function in the multi-stress response

**Figure 3:**
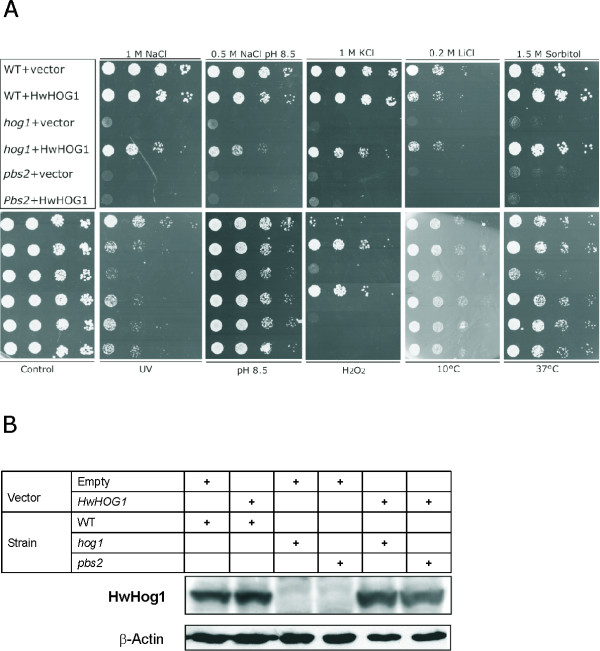
**Functional complementation of *ScHOG1 *and the multistress behaviour of transformants**. The *S. cerevisiae *wild-type (WT), *hog1 *and *pbs2 *strains were transformed with *HwHOG1*. **(A) **Ten-fold serially diluted cultures of transformed cells with empty plasmid pRD53 (vector) as control or vector carrying indicated inserts were plated onto YNB-Ura+Gal plates without salt (Control) or supplemented under different conditions (NaCl, KCl, NaCl + pH 8.5, LiCl, sorbitol, pH 8.5), as indicated. Spotted YNB-Ura+Gal plates were also assayed for growth after exposure to UV stress and at low and high temperatures. For the oxidative stress, the cultures were exposed to 50 mM H_2_O_2 _for 2 minutes prior to plating. **(B) **Immunodetection with anti-HwHog1p antibodies after SDS-PAGE electrophoresis separation of the denaturated *S. cerevisiae *cell lysates containing the indicated vectors. Immunodetection with anti-β-actin antibodies served as the sample-loading controls.

To confirm that the *HwHOG1 *gene is a true functional homologue of the yeast *HOG1 *gene, and to determine whether the activation of HwHog1 requires the MAPKK Pbs2, we carried out functional complementation assays. The *S. cerevisiae *wild-type or *hog1 *and *pbs2 *mutant strains were transformed with either the pRD53-HwHOG1 plasmid or the empty vector and selected on YNB-Ura+Gal plates supplemented with different salt and sorbitol concentrations. As shown in Figure 3A, *HwHOG1 *successfully complemented the *S. cerevisiae hog1 *phenotype, as the cells expressing HwHog1 grew only slightly slower than the *S. cerevisiae *wild-type strain on 1.0 M NaCl, 1.0 M KCl, 0.2 M LiCl and 1.5 M sorbitol plates (Fig. 3A, upper panels). These data not only demonstrate that the cells expressing HwHog1 have restored tolerance to sodium and potasium ions and to sorbitol, but also that the osmotolerance was restored only in the presence of the MAPKK Pbs2 (Fig. 3B). To check the expression levels of Hog1 and HwHog1 in transformed strains, we performed immunoblot analyses of Hog1 proteins from *S. cerevisiae *cell lysates. As shown in Figure 3B, the Hog1 protein levels were the same in all of the *HOG1*-containing strains, confirming that the effects seen are not the consequence of different amounts of the Hog1 protein in the cells, but rather the type of Hog1 expressed. We therefore conclude that the HwHog1 kinase is a true homologue of *S. cerevisiae *Hog1, and that the signal triggered by increased osmolarity transfers to Hog1 via the upstream MAPKK Pbs2, as has already been shown for the salt-sensitive *S. cerevisiae*.

Recent studies on Hog1 of *S. cerevisiae*, *C. albicans *and *Sc. pombe *have suggested that this protein can also be activated in response to heat shock, cold stress, oxidative stress, and UV injury [18,21]. To test the response of HwHog1 to these alternative stresses, we analyzed the growth of *S. cerevisiae *wild-type or *hog1 *and *pbs2 *mutant strains exposed to UV, high pH, H_2_O_2_, and low and high temperatures. As shown in Figure 3A (lower panels), HwHog1 is evidently less efficient in response to UV stress than wild-type *S. cerevisiae*. However, as has been shown previously in viability studies where both yeasts were exposed to UV irradiation, the black yeast *H. werneckii *is much more resistant to UV irradiation than *S. cerevisiae*. The highest viability of melanized *H. werneckii *cells was achieved in the stationary phase, where the melanin is most dense (personal communication, N. Gunde-Cimerman). Considering both sets of data, we can speculate that melanin is the main UV protectant in *H. werneckii*, and that activation of the HOG signalling pathway is not involved in the UV stress response.

In contrast, the HOG signalling pathway does appear to be important in oxidative stress. As shown in Figure 3A, *S. cerevisiae *expressing HwHog1 are more resistant to H_2_O_2 _than are the wild-type cells. Furthermore, this depends on the presence of the MAPKK Pbs2. The ability of *H. werneckii *to combat oxidative stress has been recently addressed, again, using hydrogen peroxide as the reactive oxygen species (ROS)-generating compound. Exposure to H_2_O_2 _resulted in a decrease in *H. werneckii *viability at extremely high salt concentrations, suggesting that the level of ROS degradation and resistance determine the upper limits of the salt tolerance of *H. werneckii *[28]. Our data here suggest that the response to oxidative stress in *H. werneckii *is also mediated via the HOG signalling pathway.

Additionally, HwHog1 appears to mediate the response to high-temperature stress (Fig. 3A, lower panels). Although wild-type *S. cerevisiae *cells did not grow better in the presence of HwHog1, *HwHOG1 *complemented the *S. cerevisiae hog1 *phenotype. Interestingly, amongst all of the stresses used in the present study, only the heat-shock response is independent of the Pbs2 protein, with growth in the *pbs2 *mutant strain being the same as in both the wild-type and the *hog1 *mutant.

These data suggest that heat-shock signals that activate HwHog1 are transmitted via a pathway distinct from the classical HOG pathway in which this MAPK and the scaffold protein Pbs2 have crucial roles. High temperature is stressful for *H. werneckii*, as has been shown by ecological studies. Recently, a novel strain of *H. werneckii *was isolated from solar salterns in the Dominican Republic. There were only a few isolates, but they showed optimal growth at 32°C [2]. Other salterns with typically lower environmental temperatures, on the other hand, are much more dominated by the black yeasts, and primarily by *H. werneckii*. These selected genotypes grew much better at lower temperatures (around 26°C), indicating that high tempertures represent additional stress for these species [3]. Activation of HwHog1 could therefore be of general importance in regulating the transcription of the gene set that is involved in combating high-temperature stress. In contrast, lower temperatures in the environment are less stressful for *H. werneckii*, and therefore in this case HwHog1 is not activated upon low-temperature exposure. The exposure of cells to elevated pH in the environment does not appear to be connected with HOG pathway activation either, as mutants with the empty vector grew as well as mutants expressing HwHog1 in conditions of high-pH stress (Fig. 3A, lower panels).

### Hog1-like MAPKs from halotolerant fungi share a high degree of similarity with orthologues of fungal origin

**Figure 4:**
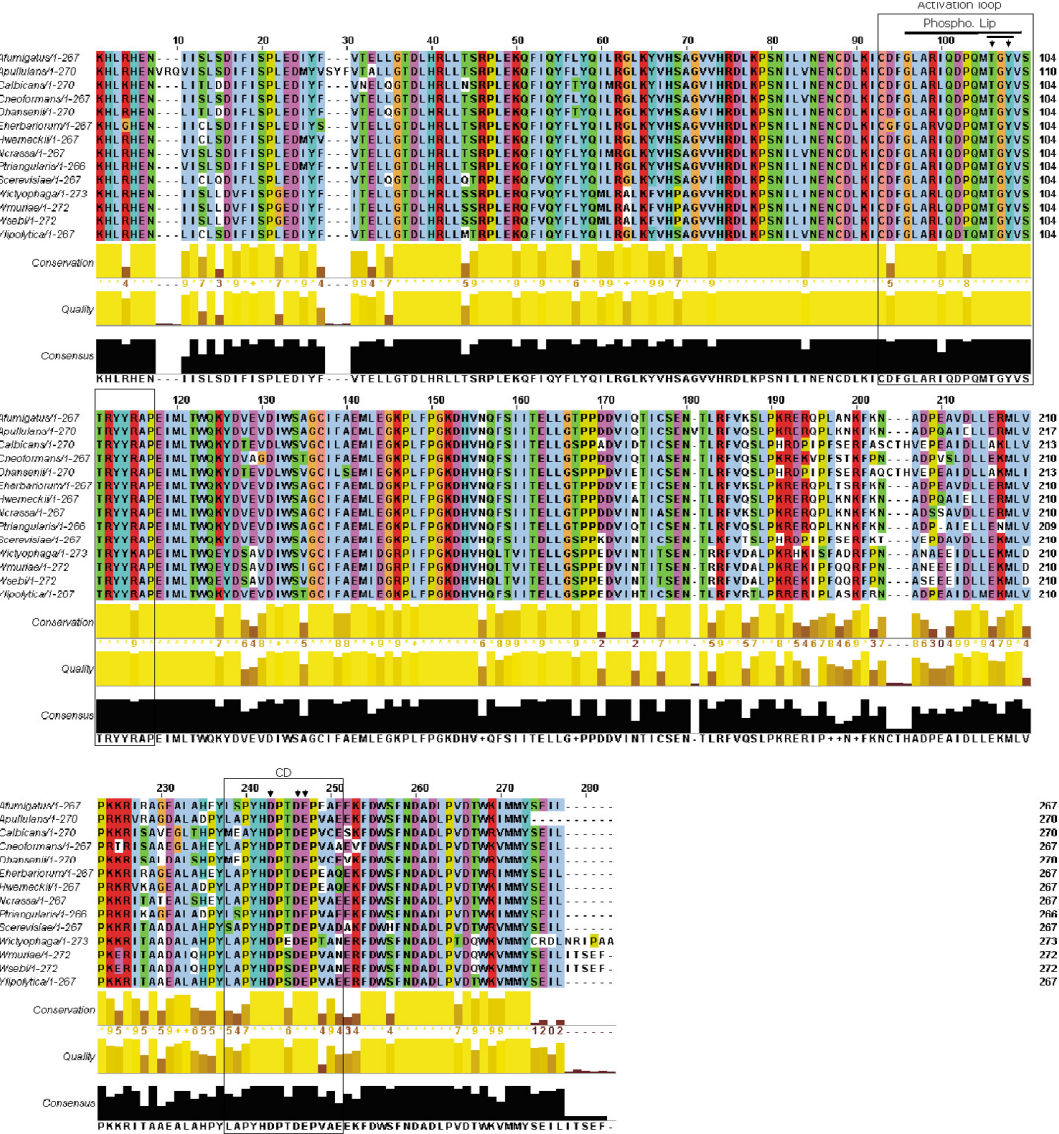
**Hog1-like MAPKs in other saltern-inhabiting halotolerant fungi**. Deduced partial amino acid sequences of Hog1-like MAPKs from the halotolerant fungi *A. pullulans, P. triangularis*, *W. ichthyophaga, W. muriae*, and *W. sebi *were aligned with the corresponding regions of the Hog1 protein from *S. cerevisiae *[GenBank: P32485], *D. hansenii *[GenBank: Q9UV50], *H. werneckii *[GenBank: AAM64214], *A. fumigatus *[GenBank: XM_747571], *C. albicans *[GenBank: Q92207], *C. neoformans *[GenBank: AAM26267], *N. crassa *[GenBank: AF297032], *Y. lipolytica *[GenBank: CAG79982], and *E. herbariorum *[GenBank: AAK25816] using the ClustalW programme. The output was graphically presented using the JalView programme implemented in ClustalW. Conservation of identical and similar amino acids is represented in conventional colours. The activation loop containing the phosphorylation Lip and the CD domain are denoted by boxes. The phosphorylation site residues of the TGY motif are indicated by black arrows.

Since very little is known about Hog1 protein orthologues from extremophilic fungi, we also sought *HOG1*-like genes in the following fungi isolated from solar salterns: the halotolerant *A. pullulans*, the halophilic *P. triangularis*, and the xerophilic *W. sebi, W. muriae*, and *W. ichthyophaga*, the last being the most halophilic fungi known to date, with an obligate requirement of at least 1.7 M NaCl [29]. Using the multiple sequence alignment function in ClustalW, HwHog1 and the translated partial Hog1 sequences from the halotolerant/halophilic fungi were aligned with Hog1 proteins from the salt-sensitive *S. cerevisiae, Aspergillus fumigatus, Cryptococcus neoformans, C. albicans, Neurospora crassa*, and *Yarrowia lipolytica*, and also the moderately halotolerant *Debaryomyces hansenii *and the halotolerant *Eurotium herbariorum *(Fig. 4). The overall alignment showed very high sequence conservation and establishes a high-quality consensus sequence of the fungal Hog1 proteins. The phosphorylation site sequence Thr-Gly-Tyr was completely conserved across all of the fungi studied (Fig. 4, black arrows), and the high conservation level extended further over the entire activation loop containing the phosphorylation Lip. The most heterogenous part of the aligned sequences preceeds the CD domain and is approximately 50 amino-acid residues in length. The region of the CD domain itself was somewhat variable, although the YHDP [T/S]DEP consensus motif contains the three essential negatively charged amino-acid residues (underlined) that are critical for the docking interactions of the MAPK with downstream effectors. Interestingly, among the fungi studied, only Hog1 from *W. ichthyophaga *was an exception regarding the separation of these three acidic amino-acid residues. In place of the Thr/Ser separator, WiHog1 contains glutamate, which results in a sequence motif that is more similar to the mammalian p38 isoforms than the fungal Hog1 orthologues. The deduced protein sequences of WmHog1 and WsHog1 were completely identical. Since large molecular distances were seen in the large subunit (LSU) rDNA sequences of *W. ichthyophaga *and *W. sebi*, and in the sequences of the rDNA internal transcribed spacers (ITS1 and ITS2) of *W. ichthyophaga *on the one hand and *W. muriae *and *W. sebi *on the other, it is possible that the genus *Wallemia *in fact comprises a complex of several phylogenetically remote genera, with the taxa in-between being extinct or not having been isolated yet [29].

**Figure 5:**
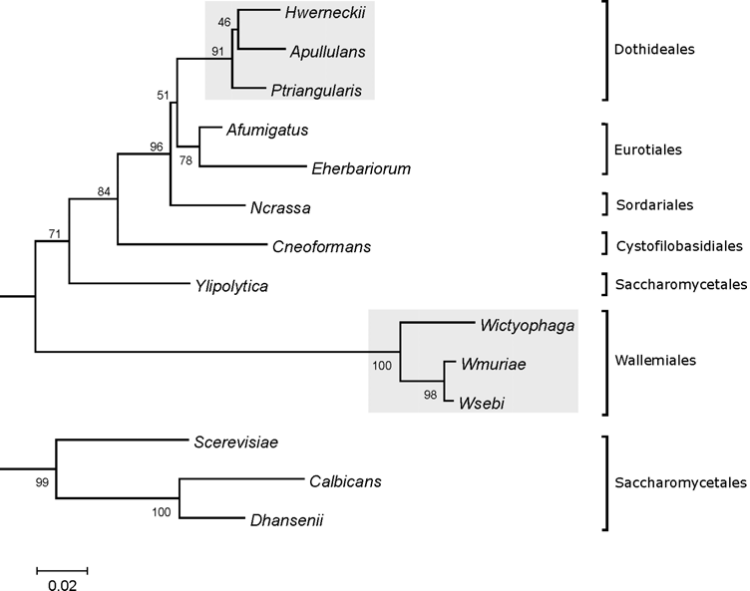
**Phylogenetic tree of deduced Hog1-like MAPKs in the halotolerant fungi and other fungal representatives**. The phylogenetic tree was constructed with the neighbour-joining method [36] based on the amino-acid sequences, using the MEGA 3.1 programme [37], and arbitrarily rooted with *W. ichthyophaga*. The numbers at the nodes are bootstrap confidence values based on 1,000 replicas. The phylogenetic tree was subjected to a Poisson correction. The saltern-inhabiting halotolerant fungi are indicated by the grey boxes.

A functional phylogenetic tree based on the amino-acid alignment of the Hog1 sequences from the above-mentioned fungi was generated using the neighbour-joining method implemented in ClustalW (Fig. 5). According to the amino-acid sequences, the fungi isolated from solar salterns are grouped into two clusters. The first is represented by halotolerant-to-extremely-halotolerant black yeasts of the order *Dothideales*. The second cluster is composed of the genus *Wallemia*, with the halophilic *W. ichthyophaga *being distinct from the more xerophilic *W. muriae *and *W. sebi*. As expected, *S. cerevisiae*, *C. albicans *and *D. hansenii *were grouped together in the *Saccharomycetales *cluster.

## Conclusion

In this study, we have demonstrated that the HOG signalling pathway has an important role in the stress response of *H. werneckii*, a highly halotolerant fungal species isolated from solar salterns. This pathway was activated not only in response to hyper-osmolar stress, but also to oxidative and heat stress, both typical of this extreme environment. Signals triggered by hyper-osmolar and oxidative stress were seen to be transmitted via the MAPKK Pbs2, the activator of the HwHog1 kinase. However, this was not the case for the high-temperature stress, in which Pbs2 appeared to have no key functional role. Our data show that HwHog1 is a true homologue of Hog1, a key MAPK of the HOG signalling pathway in *S. cerevisiae*. The 3D model of the full-length HwHog1 protein revealed an overall structural homology with other known MAPKs.

We have also shown that the Hog1-like MAPKs from other halophilic/halotolerant fungi isolated from the solar salterns share a high degree of similarity with orthologues of fungal origin. A phylogenetic tree based on the amino-acid alignment of fungal Hog1 sequences showed good agreement with the taxonomic structure of the fungi studied.

Our findings thus provide an important advance in our understanding of the response via the HOG pathway to a variety of different environmental stresses. Furthermore, this study builds on our understanding of the stress-response mechanisms in *H. werneckii*, an increasingly useful model organism for studying the mechanisms of salt tolerance in eukaryotic cells.

## Methods

### Strains and growth conditions

The cultures of *Hortaea werneckii *(MZKI B736), *Phaeotheca triangularis *(MZKI B748), *Aureobasidium pullulans *(EXF 150), *Wallemia sebi *(EXF 757), *W. muriae *(MZKI B952) and *W. ichthyophaga *(EXF 994) were from the culture collections of the Slovenian National Institute of Chemistry (MZKI) and the Department of Biology, Biotechnical Faculty, University of Ljubljana (EXF). The cells were grown at 28°C in a rotary shaker (180 rpm) in defined YNB medium, containing 0.17% (w/v) yeast nitrogen base (Qbiogene), 0.08% (w/v) complete supplement mixture (CSM) (Qbiogene), 0.5% (w/v) ammonium sulphate and 2% (w/v) glucose in deionized water, with NaCl added at the concentrations indicated, and the pH adjusted to 7.0. The cells were harvested in the mid-exponential growth phase by centrifugation at 4000 × *g *for 10 min. For the saline-stress experiments, *H. werneckii *cells were grown in YNB with 3.0 M NaCl to an optical density at 600 nm (OD_600_) of 0.7, and then the medium was replaced with either 1.0 M NaCl-YNB for the hyposaline stress, or by 4.5 M NaCl-YNB for the hypersaline stress. Aliquots of the cell suspensions were removed before the application of the stress and at 10, 30, 60, 90 and 240 min of stress. The cells were separated from the growth medium by fast filtration through a 0.45-μ m-pore filter and frozen in liquid nitrogen. The reference salt-sensitive *Saccharomyces cerevisiae *strains, the wild-type S288C strain (BY4741; Mat a; his3Δ1; leu2Δ0; met15Δ0; ura3Δ0), and the Δ*hog1 *(BY4741; Mat a; his3Δ1; leu2Δ0; met15Δ0; ura3Δ0; YLR113w::kanMX4) and Δ*pbs2 *(BY4741; Mat a; his3Δ1; leu2Δ0; met15Δ0; ura3Δ0; YJL128c::kanMX4) deletion strains were obtained from the Euroscarf Yeast Deletion Strain Collection, Germany.

### Amplification of HOG1-like genes from the genomes of the halotolerant fungi

Highly purified fungal genomic DNA was isolated from mid-exponential phase cells grown in YNB media without salt using a phenol/chloroform/isoamyl alcohol method modified for DNA isolation from filamentous fungi, as previously described [30]. Partial sequences of the *HOG1 *orthologues from the halotolerant fungi were amplified with touch-down PCR using the degenerate primers 5'-AAR CAY YTI MGI CAY GAR AA-3' and 5'-ARD ATC TCI SWR TAC ATC AT-3', corresponding to the conserved amino-acid regions in the alignment of the known Hog1 fungal orthologues. Fifty ng of genomic DNA were used as templates in 50 μL PCR reactions with Gotaq DNA polymerase (Promega). PCR was performed by 22 touch-down cycles with an annealing temperature from 55°C to 44°C, followed by 22 cycles with annealing at 44°C. The PCR products were cloned into the pGEM-T Easy vector (Promega) and sequenced. The nucleotide sequences have been deposited in the GenBank database under the following accession numbers: *PtHOG1 *[GenBank:EF158008], *ApHOG1 *[GenBank:EF158007], *WsHOG1 *[GenBank:EF158005], *WmHOG1 *[GenBank:EF158004] and *WiHOG1 *[GenBank:EF158006].

### Reverse-transcription PCR

The total RNA contents of the *H. werneckii *cells were isolated using the TRI Reagent (Sigma-Aldrich), according to the manufacturer instructions. The RNA was extracted from 200–300 mg of mid-exponential phase cells grown in salt-free YNB medium or in media with 1.0 M, 3.0 M and 4.5 M NaCl. The same protocol was used for the cells exposed to hypersaline and hyposaline shock. For the synthesis of cDNA, the RNA was first treated with DNase I (Fermentas). One μg of DNA-free RNA was used for 20 μL of the reverse transcription reaction using Superscript III reverse transcriptase (Invitrogen) and random hexamer primers (Promega), according to the manufacturer protocols.

PCR with Gotaq DNA polymerase was performed using 1 μl cDNA for the 25 μl PCR reactions with primers specific for *HwHOG1 *(5'-CGC AAA TGA CCG GTT ACG TCT CGA-3' and 5'-CAG ATC CGC ATC GTT GAA GGA CCA-3') and *H. werneckii *28S rRNA (5'-CAT CAC TGT ACT TGT TCG CTA TCG GTC-3' and 5'-GTA ACG GCG AGT GAA GCG GC-3'). *Hw28SRR *was used as an internal standard to control for variations in product abundance due to differences in individual RT-PCR reaction efficiencies. The thermal cycling was programmed for 25 cycles, each consisting of 30 s at 94°C, 30 s at 63°C and 30 s at 72°C. We empirically determined that the 25^th ^cycle lies within the optimal linear range of amplification by measuring the concentrations of the PCR products also after the 22^nd^, 24^th^, 26^th ^and 28^th ^cycles. The PCR products were resolved in agarose gels, documented by MiniBis (DNR BioImaging Systems), and the band intensities quantified with the TotalLab gel analysis programme (Non-linear Dynamics).

### Functional expression of *HwHOG1 *in *S. cerevisiae *and stress-tolerance assays

For expression of *HwHOG1 *in *S. cerevisiae*, the corresponding ORF was amplified from *H. werneckii *cDNA using the primers 5'-GGA TCC ATG GCG GAA TTC GTA CGT G-3' and 5'-GGT ACC CTA ACC GGC CAC CGT GTC AT-3' containing *BamH*I and *Kpn*I restriction sites (underlined), respectively. The resulting product was cloned into *Bam*HI/*Kpn*I sites of the pRD53 low-copy-number plasmid (*CEN*, *ARS*, *URA3*, GAL1/10 promoter, *Amp*^*R*^), resulting in the pRD53-HwHOG1 plasmid. The cloned *HwHOG1 *sequence was verified by sequencing. Yeast cells were grown overnight in YPD media at 30°C in a rotary shaker (180 rpm), to mid-exponential phase. They were then transformed with 1 μg of the pRD53-HwHOG1 construct, using the alkali-cation yeast transformation kit (Qbiogene), according to the manufacturer protocol. The transformants were selected on YNB plates without uracil (YNB-Ura). This media is the same as for YNB, except CSM lacking uracil was added instead of the CSM mix.

For the stress-tolerance assays, positive colonies were grown overnight to mid-exponential phase in YNB-Ura medium, adjusted to an OD_600 _of 0.5, 10-fold serially diluted (1–10^4 ^dilutions) with fresh medium, and spotted in 3 μL onto YNB-Ura+Gal plates supplemented as indicated (NaCl, KCl, NaCl + pH 8.5, LiCl, sorbitol, pH 8.5). The YNB-Ura+Gal plates contained 2% (w/v) galactose instead of glucose. For the UV stress, the YNB-Ura+Gal plates with spotted cultures were exposed to a UV dose of 50 J/m^2^. For the oxidative stress, the cultures were exposed to 50 mM H_2_O_2 _for 2 min prior to plating. For the cold shock, the YNB-Ura+Gal plates were incubated at 10°C, and for the heat shock, at 37°C. Otherwise the plates were incubated at 30°C for 3–5 days and the images were later acquired by scanning.

### SDS-PAGE and Western blotting

Denaturated *S. cerevisiae *cell lysates were prepared as previously described [31]. Protein concentrations were measured by spectrophotometry at 590 nm by the Bradford method with the Nanoquant reagent (Roth). Before loading, an amount of the cell fractions equal to 25 μg of protein was boiled for 5 min in 5× protein-loading buffer (Fermentas). The proteins were separated by SDS-PAGE in 10% NuPAGE polyacrylamide gels (Invitrogen) and transferred to PVDF membranes (Roth). Immunodetection with polyclonal goat anti-Hog1 antibodies (Santa Cruz Biotechnology) and polyclonal rabbit anti-goat secondary antibodies conjugated with HRP (Santa Cruz Biotechnology) were performed using the ECL detection system (Pierce).

### Homology-based 3D molecular modelling

The 3D model of HwHog1 was built by homology-based protein structure modelling with the MODELER 8v2 programme [25], which implements comparative modelling by satisfaction of spatial restraints [27]. The input was the crystallographic template structure of murine p38 [PDB: 1P38] and the HwHog1 sequence was aligned with this structure. Ten slightly different 3D models of HwHog1 with all of the non-hydrogen atoms were obtained as output. The minimum-energy 3D model of HwHog1 was chosen for the interpretation. This model was derived by minimizing violations of distance and dihedral angle constraints extracted from the template structure. The 3D model constructed passed the tests of the PROSAII [32] and PROCHECK [33,34] programmes.

## Competing interests

The author(s) declare that they have no competing interests.

## Authors' contributions

TV and ML contributed equally to this study. TV amplified the *HOG1*-like genes from the halotolerant fungi, analyzed the amino-acid alignment, constructed the phylogenetic tree, modelled the HwHog1 3D structure, constructed the pRD53-HwHog1 vector, and co-drafted the manuscript. ML carried out the functional expression studies of *HwHOG1 *in *S. cerevisiae*, performed the immunodetection of HwHog1, and co-drafted the manuscript. NGC discussed the phylogeny of selected fungi and helped to draft the manuscript. AP conceived the study, participated in its design and coordination, and helped to draft the manuscript. All of the authors have read and approved the final manuscript.
